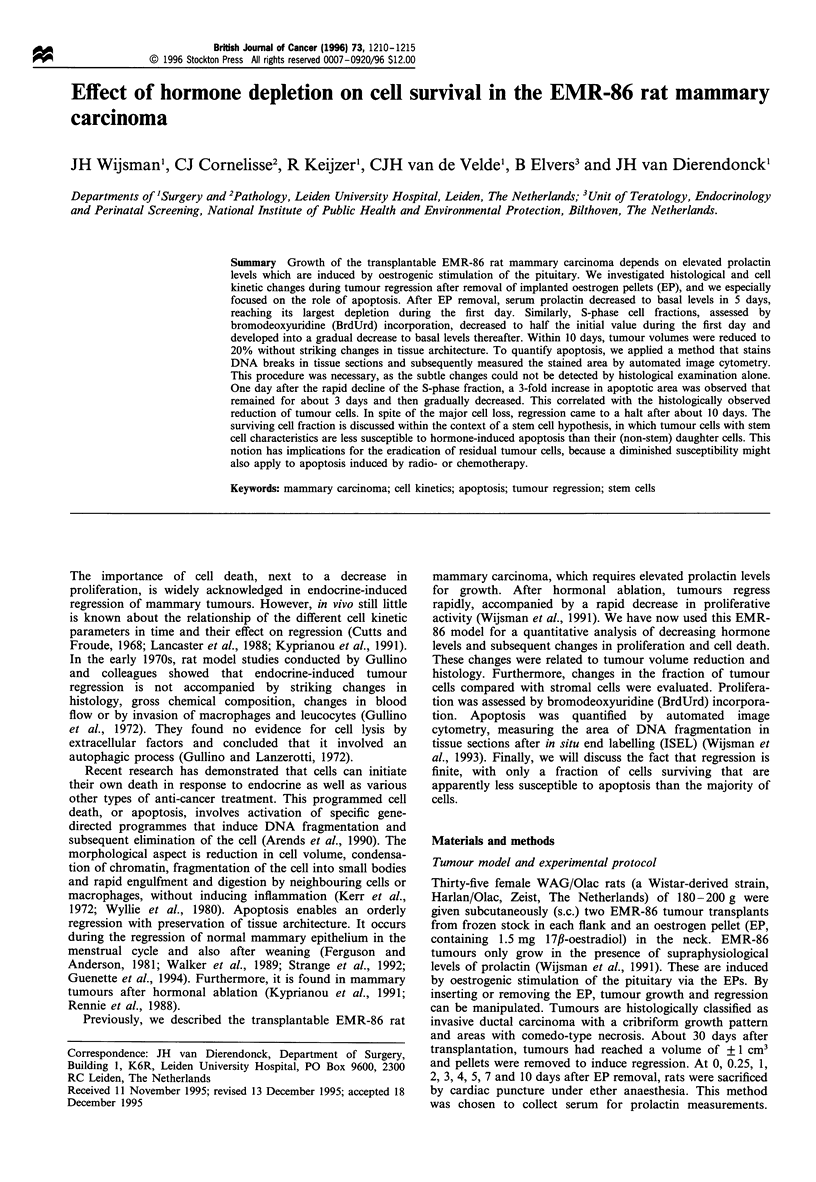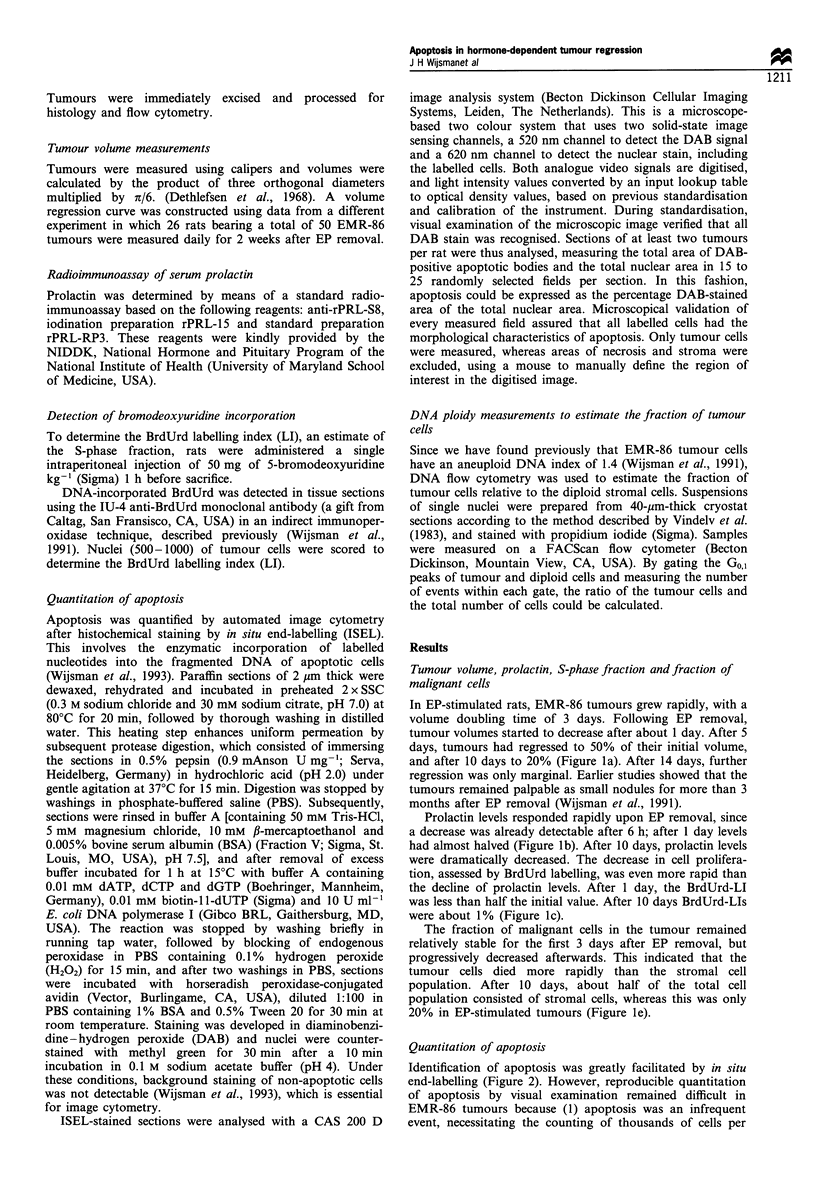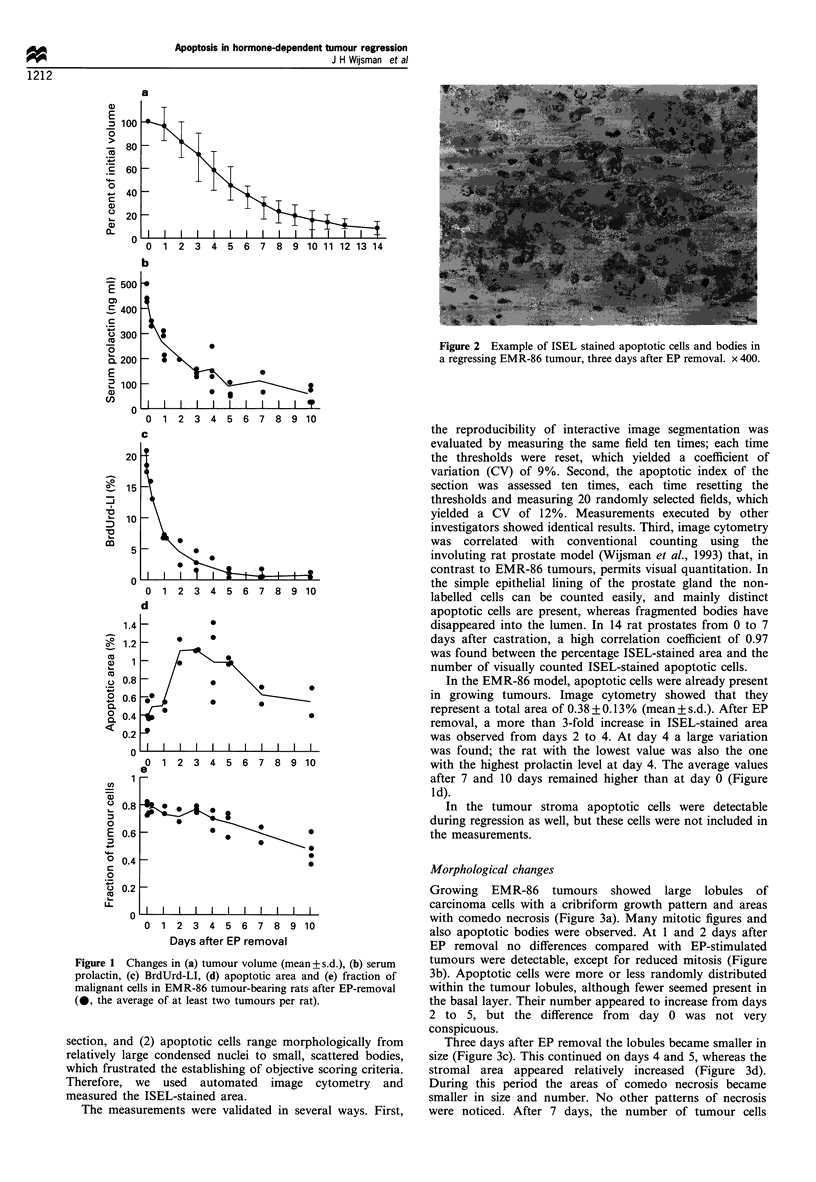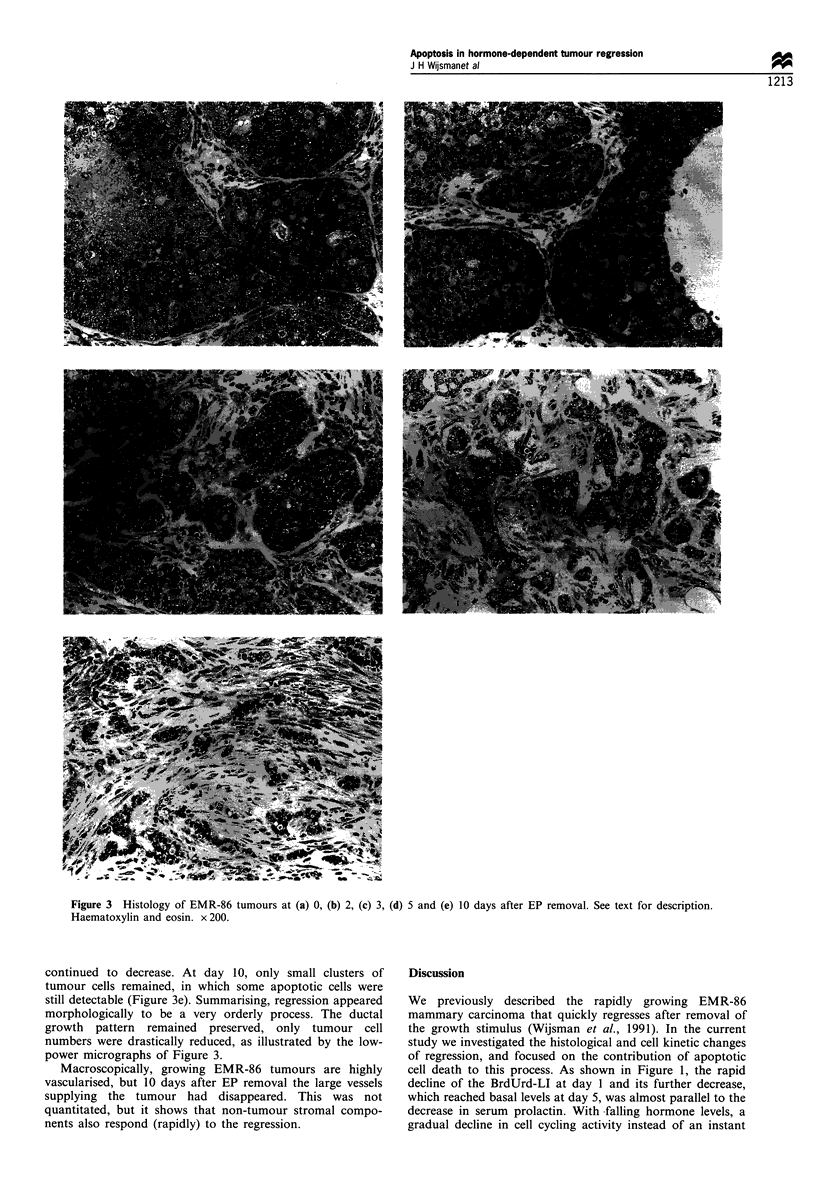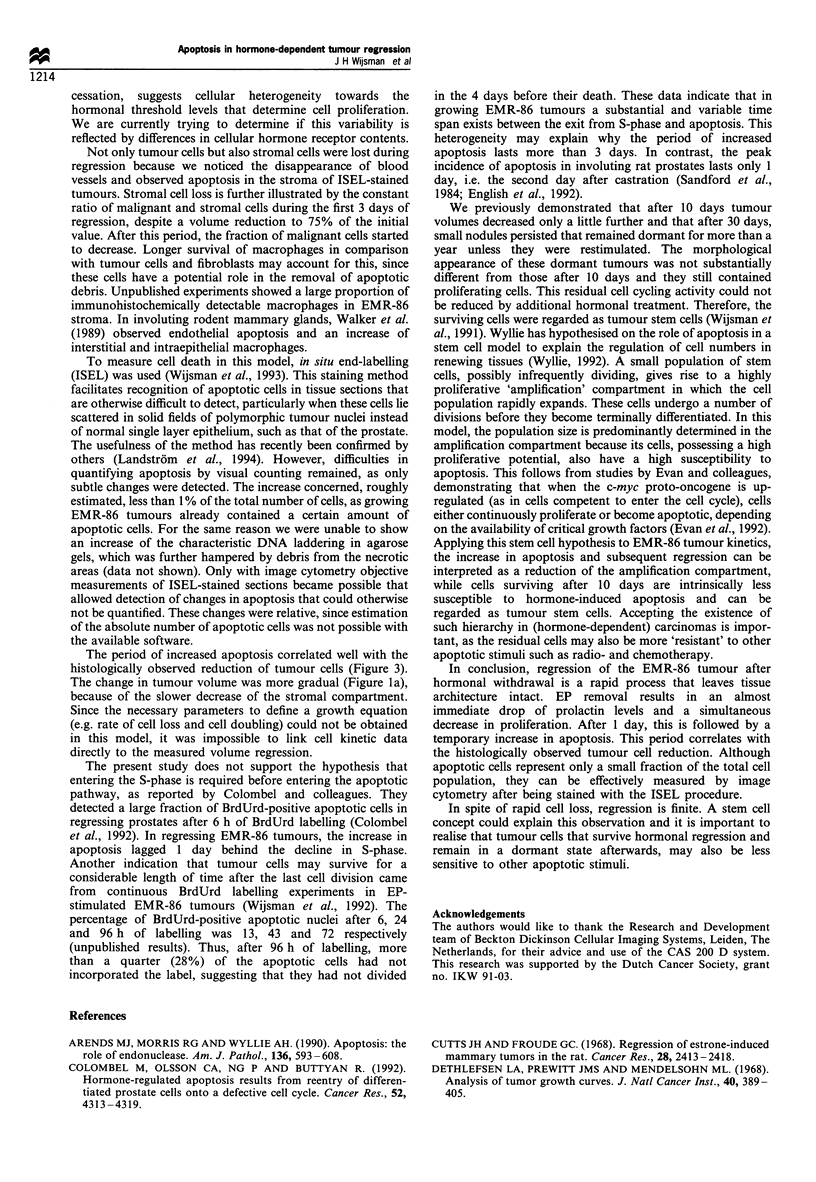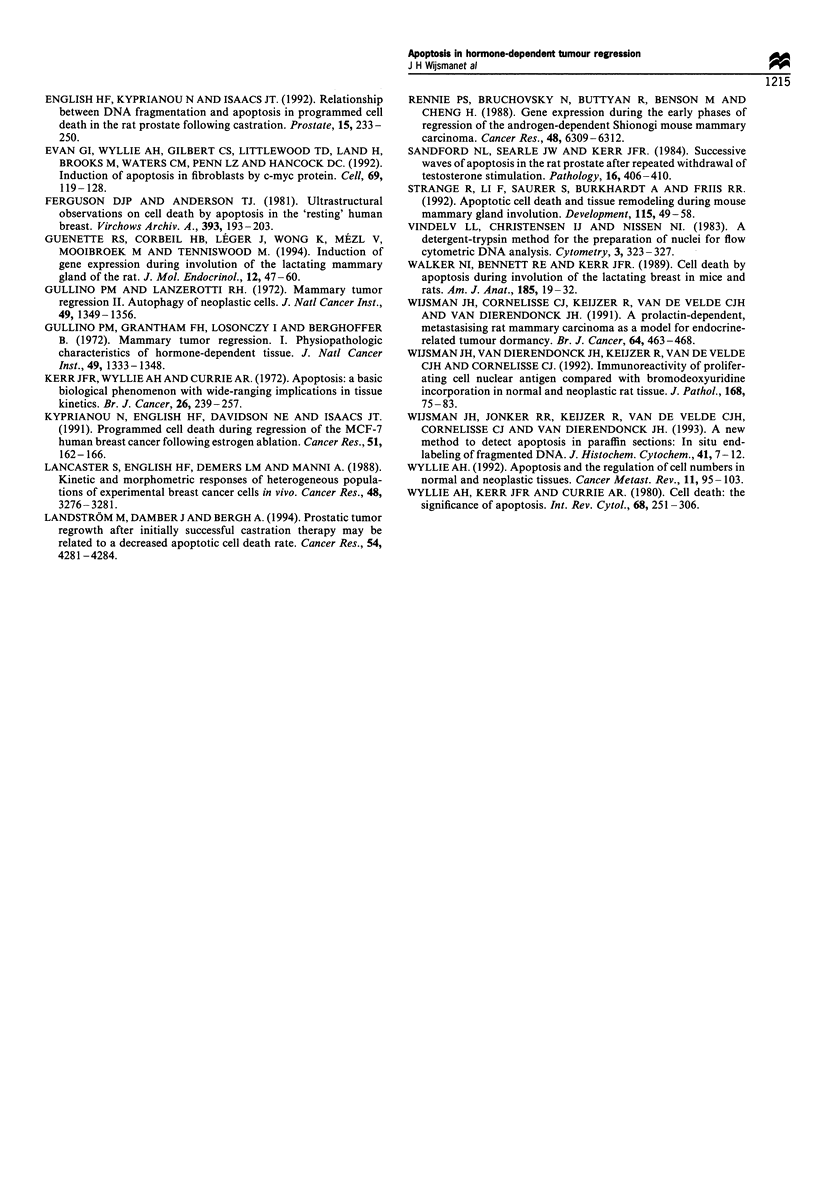# Effect of hormone depletion on cell survival in the EMR-86 rat mammary carcinoma.

**DOI:** 10.1038/bjc.1996.232

**Published:** 1996-05

**Authors:** J. H. Wijsman, C. J. Cornelisse, R. Keijzer, C. J. van de Velde, B. Elvers, J. H. van Dierendonck

**Affiliations:** Department of Surgery, Leiden University Hospital, The Netherlands.

## Abstract

**Images:**


British Journal of Cancer (1996) 73, 1210–1215
© 1996 Stockton Press All rights reserved 0007–0920/96 $12.00

### Effect of hormone depletion on cell survival in the EMR-86 rat mammary carcinoma

JH Wijsman[1], CJ Cornelisse[2], R Keijzer[1], CJH van de Velde[1], B Elvers[3] and JH van Dierendonck[1]

*Departments of [1]Surgery and [2]Pathology, Leiden University Hospital, Leiden, The Netherlands; [3]Unit of Teratology, Endocrinology and Perinatal Screening, National Institute of Public Health and Environmental Protection, Bilthoven, The Netherlands.*

**Summary** Growth of the transplantable EMR-86 rat mammary carcinoma depends on elevated prolactin levels which are induced by oestrogenic stimulation of the pituitary. We investigated histological and cell kinetic changes during tumour regression after removal of implanted oestrogen pellets (EP), and we especially focused on the role of apoptosis. After EP removal, serum prolactin decreased to basal levels in 5 days, reaching its largest depletion during the first day. Similarly, S-phase cell fractions, assessed by bromodeoxyuridine (BrdUrd) incorporation, decreased to half the initial value during the first day and developed into a gradual decrease to basal levels thereafter. Within 10 days, tumour volumes were reduced to 20% without striking changes in tissue architecture. To quantify apoptosis, we applied a method that stains DNA breaks in tissue sections and subsequently measured the stained area by automated image cytometry. This procedure was necessary, as the subtle changes could not be detected by histological examination alone. One day after the rapid decline of the S-phase fraction, a 3-fold increase in apoptotic area was observed that remained for about 3 days and then gradually decreased. This correlated with the histologically observed reduction of tumour cells. In spite of the major cell loss, regression came to a halt after about 10 days. The surviving cell fraction is discussed within the context of a stem cell hypothesis, in which tumour cells with stem cell characteristics are less susceptible to hormone-induced apoptosis than their (non-stem) daughter cells. This notion has implications for the eradication of residual tumour cells, because a diminished susceptibility might also apply to apoptosis induced by radio- or chemotherapy.

**Keywords:** mammary carcinoma; cell kinetics; apoptosis; tumour regression; stem cells

The importance of cell death, next to a decrease in proliferation, is widely acknowledged in endocrine-induced regression of mammary tumours. However, *in vivo* still little is known about the relationship of the different cell kinetic parameters in time and their effect on regression (Cutts and Froude, 1968; Lancaster *et al.*, 1988; Kyprianou *et al.*, 1991). In the early 1970s, rat model studies conducted by Gullino and colleagues showed that endocrine-induced tumour regression is not accompanied by striking changes in histology, gross chemical composition, changes in blood flow or by invasion of macrophages and leucocytes (Gullino *et al.*, 1972). They found no evidence for cell lysis by extracellular factors and concluded that it involved an autophagic process (Gullino and Lanzerotti, 1972).

Recent research has demonstrated that cells can initiate their own death in response to endocrine as well as various other types of anti-cancer treatment. This programmed cell death, or apoptosis, involves activation of specific gene-directed programmes that induce DNA fragmentation and subsequent elimination of the cell (Arends *et al.*, 1990). The morphological aspect is reduction in cell volume, condensation of chromatin, fragmentation of the cell into small bodies and rapid engulfment and digestion by neighbouring cells or macrophages, without inducing inflammation (Kerr *et al.*, 1972; Wyllie *et al.*, 1980). Apoptosis enables an orderly regression with preservation of tissue architecture. It occurs during the regression of normal mammary epithelium in the menstrual cycle and also after weaning (Ferguson and Anderson, 1981; Walker *et al.*, 1989; Strange *et al.*, 1992; Guenette *et al.*, 1994). Furthermore, it is found in mammary tumours after hormonal ablation (Kyprianou *et al.*, 1991; Rennie *et al.*, 1988).

Previously, we described the transplantable EMR-86 rat mammary carcinoma, which requires elevated prolactin levels for growth. After hormonal ablation, tumours regress rapidly, accompanied by a rapid decrease in proliferative activity (Wijsman *et al.*, 1991). We have now used this EMR-86 model for a quantitative analysis of decreasing hormone levels and subsequent changes in proliferation and cell death. These changes were related to tumour volume reduction and histology. Furthermore, changes in the fraction of tumour cells compared with stromal cells were evaluated. Proliferation was assessed by bromodeoxyuridine (BrdUrd) incorporation. Apoptosis was quantified by automated image cytometry, measuring the area of DNA fragmentation in tissue sections after *in situ* end labelling (ISEL) (Wijsman *et al.*, 1993). Finally, we will discuss the fact that regression is finite, with only a fraction of cells surviving that are apparently less susceptible to apoptosis than the majority of cells.

### Materials and methods

### *Tumour model and experimental protocol*

Thirty-five female WAG/Olac rats (a Wistar-derived strain, Harlan/Olac, Zeist, The Netherlands) of 180–200 g were given subcutaneously (s.c.) two EMR-86 tumour transplants from frozen stock in each flank and an oestrogen pellet (EP, containing 1.5 mg 17β-oestradiol) in the neck. EMR-86 tumours only grow in the presence of supraphysiological levels of prolactin (Wijsman *et al.*, 1991). These are induced by oestrogenic stimulation of the pituitary via the EPs. By inserting or removing the EP, tumour growth and regression can be manipulated. Tumours are histologically classified as invasive ductal carcinoma with a cribriform growth pattern and areas with comedo-type necrosis. About 30 days after transplantation, tumours had reached a volume of $\pm 1$ $cm^3$ and pellets were removed to induce regression. At 0, 0.25, 1, 2, 3, 4, 5, 7 and 10 days after EP removal, rats were sacrificed by cardiac puncture under ether anaesthesia. This method was chosen to collect serum for prolactin measurements.

Correspondence: JH van Dierendonck, Department of Surgery, Building 1, K6R, Leiden University Hospital, PO Box 9600, 2300 RC Leiden, The Netherlands

Received 11 November 1995; revised 13 December 1995; accepted 18 December 1995

Tumours were immediately excised and processed for histology and flow cytometry.

*Tumour volume measurements*

Tumours were measured using calipers and volumes were calculated by the product of three orthogonal diameters multiplied by $\pi/6$. (Dethlefsen *et al.*, 1968). A volume regression curve was constructed using data from a different experiment in which 26 rats bearing a total of 50 EMR-86 tumours were measured daily for 2 weeks after EP removal.

*Radioimmunoassay of serum prolactin*

Prolactin was determined by means of a standard radioimmunoassay based on the following reagents: anti-rPRL-S8, iodination preparation rPRL-15 and standard preparation rPRL-RP3. These reagents were kindly provided by the NIDDK, National Hormone and Pituitary Program of the National Institute of Health (University of Maryland School of Medicine, USA).

*Detection of bromodeoxyuridine incorporation*

To determine the BrdUrd labelling index (LI), an estimate of the S-phase fraction, rats were administered a single intraperitoneal injection of 50 mg of 5-bromodeoxyuridine $kg^{-1}$ (Sigma) 1 h before sacrifice.

DNA-incorporated BrdUrd was detected in tissue sections using the IU-4 anti-BrdUrd monoclonal antibody (a gift from Caltag, San Fransisco, CA, USA) in an indirect immunoperoxidase technique, described previously (Wijsman *et al.*, 1991). Nuclei (500–1000) of tumour cells were scored to determine the BrdUrd labelling index (LI).

*Quantitation of apoptosis*

Apoptosis was quantified by automated image cytometry after histochemical staining by *in situ* end-labelling (ISEL). This involves the enzymatic incorporation of labelled nucleotides into the fragmented DNA of apoptotic cells (Wijsman *et al.*, 1993). Paraffin sections of 2 $\mu$m thick were dewaxed, rehydrated and incubated in preheated 2 × SSC (0.3 M sodium chloride and 30 mM sodium citrate, pH 7.0) at 80°C for 20 min, followed by thorough washing in distilled water. This heating step enhances uniform permeation by subsequent protease digestion, which consisted of immersing the sections in 0.5% pepsin (0.9 mAnson U $mg^{-1}$; Serva, Heidelberg, Germany) in hydrochloric acid (pH 2.0) under gentle agitation at 37°C for 15 min. Digestion was stopped by washings in phosphate-buffered saline (PBS). Subsequently, sections were rinsed in buffer A [containing 50 mM Tris-HCl, 5 mM magnesium chloride, 10 mM $\beta$-mercaptoethanol and 0.005% bovine serum albumin (BSA) (Fraction V; Sigma, St. Louis, MO, USA), pH 7.5], and after removal of excess buffer incubated for 1 h at 15°C with buffer A containing 0.01 mM dATP, dCTP and dGTP (Boehringer, Mannheim, Germany), 0.01 mM biotin-11-dUTP (Sigma) and 10 U $ml^{-1}$ *E. coli* DNA polymerase I (Gibco BRL, Gaithersburg, MD, USA). The reaction was stopped by washing briefly in running tap water, followed by blocking of endogenous peroxidase in PBS containing 0.1% hydrogen peroxide ($H_2O_2$) for 15 min, and after two washings in PBS, sections were incubated with horseradish peroxidase-conjugated avidin (Vector, Burlingame, CA, USA), diluted 1:100 in PBS containing 1% BSA and 0.5% Tween 20 for 30 min at room temperature. Staining was developed in diaminobenzidine–hydrogen peroxide (DAB) and nuclei were counterstained with methyl green for 30 min after a 10 min incubation in 0.1 M sodium acetate buffer (pH 4). Under these conditions, background staining of non-apoptotic cells was not detectable (Wijsman *et al.*, 1993), which is essential for image cytometry.

ISEL-stained sections were analysed with a CAS 200 D image analysis system (Becton Dickinson Cellular Imaging Systems, Leiden, The Netherlands). This is a microscope-based two colour system that uses two solid-state image sensing channels, a 520 nm channel to detect the DAB signal and a 620 nm channel to detect the nuclear stain, including the labelled cells. Both analogue video signals are digitised, and light intensity values converted by an input lookup table to optical density values, based on previous standardisation and calibration of the instrument. During standardisation, visual examination of the microscopic image verified that all DAB stain was recognised. Sections of at least two tumours per rat were thus analysed, measuring the total area of DAB-positive apoptotic bodies and the total nuclear area in 15 to 25 randomly selected fields per section. In this fashion, apoptosis could be expressed as the percentage DAB-stained area of the total nuclear area. Microscopical validation of every measured field assured that all labelled cells had the morphological characteristics of apoptosis. Only tumour cells were measured, whereas areas of necrosis and stroma were excluded, using a mouse to manually define the region of interest in the digitised image.

*DNA ploidy measurements to estimate the fraction of tumour cells*

Since we have found previously that EMR-86 tumour cells have an aneuploid DNA index of 1.4 (Wijsman *et al.*, 1991), DNA flow cytometry was used to estimate the fraction of tumour cells relative to the diploid stromal cells. Suspensions of single nuclei were prepared from 40-$\mu$m-thick cryostat sections according to the method described by Vindelv *et al.* (1983), and stained with propidium iodide (Sigma). Samples were measured on a FACScan flow cytometer (Becton Dickinson, Mountain View, CA, USA). By gating the $G_{0,1}$ peaks of tumour and diploid cells and measuring the number of events within each gate, the ratio of the tumour cells and the total number of cells could be calculated.

### Results

*Tumour volume, prolactin, S-phase fraction and fraction of malignant cells*

In EP-stimulated rats, EMR-86 tumours grew rapidly, with a volume doubling time of 3 days. Following EP removal, tumour volumes started to decrease after about 1 day. After 5 days, tumours had regressed to 50% of their initial volume, and after 10 days to 20% (Figure 1a). After 14 days, further regression was only marginal. Earlier studies showed that the tumours remained palpable as small nodules for more than 3 months after EP removal (Wijsman *et al.*, 1991).

Prolactin levels responded rapidly upon EP removal, since a decrease was already detectable after 6 h; after 1 day levels had almost halved (Figure 1b). After 10 days, prolactin levels were dramatically decreased. The decrease in cell proliferation, assessed by BrdUrd labelling, was even more rapid than the decline of prolactin levels. After 1 day, the BrdUrd-LI was less than half the initial value. After 10 days BrdUrd-LIs were about 1% (Figure 1c).

The fraction of malignant cells in the tumour remained relatively stable for the first 3 days after EP removal, but progressively decreased afterwards. This indicated that the tumour cells died more rapidly than the stromal cell population. After 10 days, about half of the total cell population consisted of stromal cells, whereas this was only 20% in EP-stimulated tumours (Figure 1e).

*Quantitation of apoptosis*

Identification of apoptosis was greatly facilitated by *in situ* end-labelling (Figure 2). However, reproducible quantitation of apoptosis by visual examination remained difficult in EMR-86 tumours because (1) apoptosis was an infrequent event, necessitating the counting of thousands of cells per

**Figure 1** Changes in (**a**) tumour volume (mean ± s.d.), (**b**) serum prolactin, (**c**) BrdUrd-LI, (**d**) apoptotic area and (**e**) fraction of malignant cells in EMR-86 tumour-bearing rats after EP-removal (●, the average of at least two tumours per rat).

section, and (2) apoptotic cells range morphologically from relatively large condensed nuclei to small, scattered bodies, which frustrated the establishing of objective scoring criteria. Therefore, we used automated image cytometry and measured the ISEL-stained area.

The measurements were validated in several ways. First,

**Figure 2** Example of ISEL stained apoptotic cells and bodies in a regressing EMR-86 tumour, three days after EP removal. ×400.

the reproducibility of interactive image segmentation was evaluated by measuring the same field ten times; each time the thresholds were reset, which yielded a coefficient of variation (CV) of 9%. Second, the apoptotic index of the section was assessed ten times, each time resetting the thresholds and measuring 20 randomly selected fields, which yielded a CV of 12%. Measurements executed by other investigators showed identical results. Third, image cytometry was correlated with conventional counting using the involuting rat prostate model (Wijsman *et al*., 1993) that, in contrast to EMR-86 tumours, permits visual quantitation. In the simple epithelial lining of the prostate gland the non-labelled cells can be counted easily, and mainly distinct apoptotic cells are present, whereas fragmented bodies have disappeared into the lumen. In 14 rat prostates from 0 to 7 days after castration, a high correlation coefficient of 0.97 was found between the percentage ISEL-stained area and the number of visually counted ISEL-stained apoptotic cells.

In the EMR-86 model, apoptotic cells were already present in growing tumours. Image cytometry showed that they represent a total area of 0.38 ± 0.13% (mean ± s.d.). After EP removal, a more than 3-fold increase in ISEL-stained area was observed from days 2 to 4. At day 4 a large variation was found; the rat with the lowest value was also the one with the highest prolactin level at day 4. The average values after 7 and 10 days remained higher than at day 0 (Figure 1d).

In the tumour stroma apoptotic cells were detectable during regression as well, but these cells were not included in the measurements.

### *Morphological changes*

Growing EMR-86 tumours showed large lobules of carcinoma cells with a cribriform growth pattern and areas with comedo necrosis (Figure 3a). Many mitotic figures and also apoptotic bodies were observed. At 1 and 2 days after EP removal no differences compared with EP-stimulated tumours were detectable, except for reduced mitosis (Figure 3b). Apoptotic cells were more or less randomly distributed within the tumour lobules, although fewer seemed present in the basal layer. Their number appeared to increase from days 2 to 5, but the difference from day 0 was not very conspicuous.

Three days after EP removal the lobules became smaller in size (Figure 3c). This continued on days 4 and 5, whereas the stromal area appeared relatively increased (Figure 3d). During this period the areas of comedo necrosis became smaller in size and number. No other patterns of necrosis were noticed. After 7 days, the number of tumour cells

**Figure 3** Histology of EMR-86 tumours at (**a**) 0, (**b**) 2, (**c**) 3, (**d**) 5 and (**e**) 10 days after EP removal. See text for description. Haematoxylin and eosin. ×200.

continued to decrease. At day 10, only small clusters of tumour cells remained, in which some apoptotic cells were still detectable (Figure 3e). Summarising, regression appeared morphologically to be a very orderly process. The ductal growth pattern remained preserved, only tumour cell numbers were drastically reduced, as illustrated by the low-power micrographs of Figure 3.

Macroscopically, growing EMR-86 tumours are highly vascularised, but 10 days after EP removal the large vessels supplying the tumour had disappeared. This was not quantitated, but it shows that non-tumour stromal components also respond (rapidly) to the regression.

### Discussion

We previously described the rapidly growing EMR-86 mammary carcinoma that quickly regresses after removal of the growth stimulus (Wijsman *et al.*, 1991). In the current study we investigated the histological and cell kinetic changes of regression, and focused on the contribution of apoptotic cell death to this process. As shown in Figure 1, the rapid decline of the BrdUrd-LI at day 1 and its further decrease, which reached basal levels at day 5, was almost parallel to the decrease in serum prolactin. With falling hormone levels, a gradual decline in cell cycling activity instead of an instant cessation, suggests cellular heterogeneity towards the hormonal threshold levels that determine cell proliferation. We are currently trying to determine if this variability is reflected by differences in cellular hormone receptor contents.

Not only tumour cells but also stromal cells were lost during regression because we noticed the disappearance of blood vessels and observed apoptosis in the stroma of ISEL-stained tumours. Stromal cell loss is further illustrated by the constant ratio of malignant and stromal cells during the first 3 days of regression, despite a volume reduction to 75% of the initial value. After this period, the fraction of malignant cells started to decrease. Longer survival of macrophages in comparison with tumour cells and fibroblasts may account for this, since these cells have a potential role in the removal of apoptotic debris. Unpublished experiments showed a large proportion of immunohistochemically detectable macrophages in EMR-86 stroma. In involuting rodent mammary glands, Walker *et al.* (1989) observed endothelial apoptosis and an increase of interstitial and intraepithelial macrophages.

To measure cell death in this model, *in situ* end-labelling (ISEL) was used (Wijsman *et al.*, 1993). This staining method facilitates recognition of apoptotic cells in tissue sections that are otherwise difficult to detect, particularly when these cells lie scattered in solid fields of polymorphic tumour nuclei instead of normal single layer epithelium, such as that of the prostate. The usefulness of the method has recently been confirmed by others (Landström *et al.*, 1994). However, difficulties in quantifying apoptosis by visual counting remained, as only subtle changes were detected. The increase concerned, roughly estimated, less than 1% of the total number of cells, as growing EMR-86 tumours already contained a certain amount of apoptotic cells. For the same reason we were unable to show an increase of the characteristic DNA laddering in agarose gels, which was further hampered by debris from the necrotic areas (data not shown). Only with image cytometry objective measurements of ISEL-stained sections became possible that allowed detection of changes in apoptosis that could otherwise not be quantified. These changes were relative, since estimation of the absolute number of apoptotic cells was not possible with the available software.

The period of increased apoptosis correlated well with the histologically observed reduction of tumour cells (Figure 3). The change in tumour volume was more gradual (Figure 1a), because of the slower decrease of the stromal compartment. Since the necessary parameters to define a growth equation (e.g. rate of cell loss and cell doubling) could not be obtained in this model, it was impossible to link cell kinetic data directly to the measured volume regression.

The present study does not support the hypothesis that entering the S-phase is required before entering the apoptotic pathway, as reported by Colombel and colleagues. They detected a large fraction of BrdUrd-positive apoptotic cells in regressing prostates after 6 h of BrdUrd labelling (Colombel *et al.*, 1992). In regressing EMR-86 tumours, the increase in apoptosis lagged 1 day behind the decline in S-phase. Another indication that tumour cells may survive for a considerable length of time after the last cell division came from continuous BrdUrd labelling experiments in EP-stimulated EMR-86 tumours (Wijsman *et al.*, 1992). The percentage of BrdUrd-positive apoptotic nuclei after 6, 24 and 96 h of labelling was 13, 43 and 72 respectively (unpublished results). Thus, after 96 h of labelling, more than a quarter (28%) of the apoptotic cells had not incorporated the label, suggesting that they had not divided in the 4 days before their death. These data indicate that in growing EMR-86 tumours a substantial and variable time span exists between the exit from S-phase and apoptosis. This heterogeneity may explain why the period of increased apoptosis lasts more than 3 days. In contrast, the peak incidence of apoptosis in involuting rat prostates lasts only 1 day, i.e. the second day after castration (Sandford *et al.*, 1984; English *et al.*, 1992).

We previously demonstrated that after 10 days tumour volumes decreased only a little further and that after 30 days, small nodules persisted that remained dormant for more than a year unless they were restimulated. The morphological appearance of these dormant tumours was not substantially different from those after 10 days and they still contained proliferating cells. This residual cell cycling activity could not be reduced by additional hormonal treatment. Therefore, the surviving cells were regarded as tumour stem cells (Wijsman *et al.*, 1991). Wyllie has hypothesised on the role of apoptosis in a stem cell model to explain the regulation of cell numbers in renewing tissues (Wyllie, 1992). A small population of stem cells, possibly infrequently dividing, gives rise to a highly proliferative 'amplification' compartment in which the cell population rapidly expands. These cells undergo a number of divisions before they become terminally differentiated. In this model, the population size is predominantly determined in the amplification compartment because its cells, possessing a high proliferative potential, also have a high susceptibility to apoptosis. This follows from studies by Evan and colleagues, demonstrating that when the c-*myc* proto-oncogene is up-regulated (as in cells competent to enter the cell cycle), cells either continuously proliferate or become apoptotic, depending on the availability of critical growth factors (Evan *et al.*, 1992). Applying this stem cell hypothesis to EMR-86 tumour kinetics, the increase in apoptosis and subsequent regression can be interpreted as a reduction of the amplification compartment, while cells surviving after 10 days are intrinsically less susceptible to hormone-induced apoptosis and can be regarded as tumour stem cells. Accepting the existence of such hierarchy in (hormone-dependent) carcinomas is important, as the residual cells may also be more 'resistant' to other apoptotic stimuli such as radio- and chemotherapy.

In conclusion, regression of the EMR-86 tumour after hormonal withdrawal is a rapid process that leaves tissue architecture intact. EP removal results in an almost immediate drop of prolactin levels and a simultaneous decrease in proliferation. After 1 day, this is followed by a temporary increase in apoptosis. This period correlates with the histologically observed tumour cell reduction. Although apoptotic cells represent only a small fraction of the total cell population, they can be effectively measured by image cytometry after being stained with the ISEL procedure.

In spite of rapid cell loss, regression is finite. A stem cell concept could explain this observation and it is important to realise that tumour cells that survive hormonal regression and remain in a dormant state afterwards, may also be less sensitive to other apoptotic stimuli.

**Acknowledgements**
The authors would like to thank the Research and Development team of Beckton Dickinson Cellular Imaging Systems, Leiden, The Netherlands, for their advice and use of the CAS 200 D system. This research was supported by the Dutch Cancer Society, grant no. IKW 91-03.

### References

ARENDS MJ, MORRIS RG AND WYLLIE AH. (1990). Apoptosis: the role of endonuclease. *Am. J. Pathol.*, **136**, 593–608.

COLOMBEL M, OLSSON CA, NG P AND BUTTYAN R. (1992). Hormone-regulated apoptosis results from reentry of differentiated prostate cells onto a defective cell cycle. *Cancer Res.*, **52**, 4313–4319.

CUTTS JH AND FROUDE GC. (1968). Regression of estrone-induced mammary tumors in the rat. *Cancer Res.*, **28**, 2413–2418.

DETHLEFSEN LA, PREWITT JMS AND MENDELSOHN ML. (1968). Analysis of tumor growth curves. *J. Natl Cancer Inst.*, **40**, 389–405.

ENGLISH HF, KYPRIANOU N AND ISAACS JT. (1992). Relationship between DNA fragmentation and apoptosis in programmed cell death in the rat prostate following castration. *Prostate*, **15**, 233–250.

EVAN GI, WYLLIE AH, GILBERT CS, LITTLEWOOD TD, LAND H, BROOKS M, WATERS CM, PENN LZ AND HANCOCK DC. (1992). Induction of apoptosis in fibroblasts by c-myc protein. *Cell*, **69**, 119–128.

FERGUSON DJP AND ANDERSON TJ. (1981). Ultrastructural observations on cell death by apoptosis in the 'resting' human breast. *Virchows Archiv. A.*, **393**, 193–203.

GUENETTE RS, CORBEIL HB, LÉGER J, WONG K, MÉZL V, MOOIBROEK M AND TENNISWOOD M. (1994). Induction of gene expression during involution of the lactating mammary gland of the rat. *J. Mol. Endocrinol.*, **12**, 47–60.

GULLINO PM AND LANZEROTTI RH. (1972). Mammary tumor regression II. Autophagy of neoplastic cells. *J. Natl Cancer Inst.*, **49**, 1349–1356.

GULLINO PM, GRANTHAM FH, LOSONCZY I AND BERGHOFFER B. (1972). Mammary tumor regression. I. Physiopathologic characteristics of hormone-dependent tissue. *J. Natl Cancer Inst.*, **49**, 1333–1348.

KERR JFR, WYLLIE AH AND CURRIE AR. (1972). Apoptosis: a basic biological phenomenon with wide-ranging implications in tissue kinetics. *Br. J. Cancer*, **26**, 239–257.

KYPRIANOU N, ENGLISH HF, DAVIDSON NE AND ISAACS JT. (1991). Programmed cell death during regression of the MCF-7 human breast cancer following estrogen ablation. *Cancer Res.*, **51**, 162–166.

LANCASTER S, ENGLISH HF, DEMERS LM AND MANNI A. (1988). Kinetic and morphometric responses of heterogeneous populations of experimental breast cancer cells *in vivo*. *Cancer Res.*, **48**, 3276–3281.

LANDSTRÖM M, DAMBER J AND BERGH A. (1994). Prostatic tumor regrowth after initially successful castration therapy may be related to a decreased apoptotic cell death rate. *Cancer Res.*, **54**, 4281–4284.

RENNIE PS, BRUCHOVSKY N, BUTTYAN R, BENSON M AND CHENG H. (1988). Gene expression during the early phases of regression of the androgen-dependent Shionogi mouse mammary carcinoma. *Cancer Res.*, **48**, 6309–6312.

SANDFORD NL, SEARLE JW AND KERR JFR. (1984). Successive waves of apoptosis in the rat prostate after repeated withdrawal of testosterone stimulation. *Pathology*, **16**, 406–410.

STRANGE R, LI F, SAURER S, BURKHARDT A AND FRIIS RR. (1992). Apoptotic cell death and tissue remodeling during mouse mammary gland involution. *Development*, **115**, 49–58.

VINDELV LL, CHRISTENSEN IJ AND NISSEN NI. (1983). A detergent-trypsin method for the preparation of nuclei for flow cytometric DNA analysis. *Cytometry*, **3**, 323–327.

WALKER NI, BENNETT RE AND KERR JFR. (1989). Cell death by apoptosis during involution of the lactating breast in mice and rats. *Am. J. Anat.*, **185**, 19–32.

WIJSMAN JH, CORNELISSE CJ, KEIJZER R, VAN DE VELDE CJH AND VAN DIERENDONCK JH. (1991). A prolactin-dependent, metastasising rat mammary carcinoma as a model for endocrine-related tumour dormancy. *Br. J. Cancer*, **64**, 463–468.

WIJSMAN JH, VAN DIERENDONCK JH, KEIJZER R, VAN DE VELDE CJH AND CORNELISSE CJ. (1992). Immunoreactivity of proliferating cell nuclear antigen compared with bromodeoxyuridine incorporation in normal and neoplastic rat tissue. *J. Pathol.*, **168**, 75–83.

WIJSMAN JH, JONKER RR, KEIJZER R, VAN DE VELDE CJH, CORNELISSE CJ AND VAN DIERENDONCK JH. (1993). A new method to detect apoptosis in paraffin sections: In situ end-labeling of fragmented DNA. *J. Histochem. Cytochem.*, **41**, 7–12.

WYLLIE AH. (1992). Apoptosis and the regulation of cell numbers in normal and neoplastic tissues. *Cancer Metast. Rev.*, **11**, 95–103.

WYLLIE AH, KERR JFR AND CURRIE AR. (1980). Cell death: the significance of apoptosis. *Int. Rev. Cytol.*, **68**, 251–306.